# Distinct drivers of stadium attendance and online streaming: evidence from the Chinese women's super league

**DOI:** 10.3389/fspor.2025.1695179

**Published:** 2025-11-13

**Authors:** Tiantian Liu, Shanshan Li, Zhiyu Han, Huayong Niu

**Affiliations:** 1 International Business School, Beijing Foreign Studies University, Beijing, China; 2 School of Sports Science and Physical Education, Nanjing Normal University, Nanjing, China; 3 Belarusian State University of Physical Education and Sport, Minsk, Belarus

**Keywords:** women's football, attendance demand, online streaming demand, competitive balance, digital consumption in sport, artificial intelligence

## Abstract

**Introduction:**

This study investigates the determinants of women's football viewership by comparing stadium attendance and online streaming demand in the Chinese Women's Super League during the 2023 season.

**Methods:**

Match-level data were analyzed using ordinary least squares regression for 125 games with attendance records and negative binomial regression for 126 games with streaming data.

**Results:**

The results revealed distinct drivers across the two forms of consumption. Higher stadium attendance was associated with the home team's historical championships, lower per capita disposable income in the host city, and weekend scheduling. In contrast, online streaming demand was shaped by competitive balance and team quality, as well as seasonal variation and the length of time replay videos remained accessible.

**Discussion:**

These findings demonstrate that live attendance is influenced primarily by structural and contextual factors, whereas digital audiences respond more directly to match competitiveness and content availability. The study highlights the complementary nature of in-person and digital engagement in women's football and underscores the growing role of data-driven approaches in advancing audience research. By integrating audience behavior analysis with innovations in digital technology, wearable monitoring, and artificial intelligence, this research contributes to the development of interdisciplinary frameworks that can enhance fan engagement, expand the visibility of women's football, and support broader applications of sports science in the digital era.

## 1 Introduction

Women's football has a long and rich history, yet it continues to struggle to achieve the same visibility and recognition as the men's game (1). Despite its deep cultural roots and the undeniable talent of its players, structural challenges such as limited funding, insufficient media exposure, and persistent cultural barriers have slowed its global development. These disparities have contributed to an enduring gap between women's and men's football in terms of professionalization and audience engagement. Nevertheless, women's football has proven to be an increasingly attractive product, supported by the skill, passion, and commitment of its athletes (2). In recent years, this growing appeal has been amplified by new forms of digital media, creating opportunities to reach broader audiences and foster stronger fan engagement (3).

Before the outbreak of the COVID-19 pandemic, stadium attendance was a central source of revenue for professional clubs and leagues worldwide. The atmosphere of live events, combined with direct fan participation, generated unique experiences that supported financial sustainability through ticket sales, merchandise, and concessions (4). The pandemic, however, brought unprecedented disruptions: lockdowns, social distancing measures, and public health restrictions suspended competitions and closed stadiums. These conditions forced sports organizations to explore alternative channels to maintain audience engagement, accelerating the shift toward digital platforms and live streaming as complementary forms of consumption.

Women's football has consistently faced challenges in attracting large in-person audiences, and media coverage of women's matches remains limited even on a global scale (5). The COVID-19 pandemic further intensified these difficulties by reducing visibility and weakening audience support at a time when women's sport was already striving for broader recognition. Although significant efforts have been made to promote and develop women's football, the pandemic underscored the structural disparities in exposure and engagement when compared with men's competitions. At the same time, it highlighted the urgent need to expand alternative channels of consumption, particularly digital streaming, to sustain audience interest and ensure continued growth.

Sports organizations have increasingly recognized the potential of digital platforms to broaden their audiences by extending traditional broadcast services. Platforms such as Amazon's Twitch, initially established for gaming but now attracting over a million daily viewers, have become prominent channels for live sports content. Major leagues such as the NFL and NBA were among the first to complement television coverage with streaming services, setting a precedent that has since been followed by other competitions (6–8). While the popularity of these platforms has grown rapidly (9), empirical research on how audiences choose and experience online viewing options in sport remains limited, particularly in women's football.

The pandemic not only intensified existing challenges but also created new opportunities for women's football (10). With stadiums closed, online streaming platforms became a key channel for expanding the sport's reach to wider audiences. International competitions such as the FIFA Women's World Cup and the UEFA Women's Champions League demonstrated the strong appeal and growth potential of women's football by drawing substantial online viewership and heightened global attention (11).

As the effects of the pandemic subsided and restrictions were lifted, stadiums reopened and fans returned to the live atmosphere of sporting events. This resurgence of in-person attendance restored both enthusiasm and revenues for sports organizations (12). At the same time, online streaming did not decline but continued to expand, reflecting a lasting transformation in consumption habits (8, 13). The pandemic thus acted as a catalyst for long-term change, with streaming services now reaching broader audiences and fostering stronger engagement than before (14).

Overall, the pandemic fundamentally altered the way sports were consumed, reinforcing the growing importance of digital engagement. During the COVID-19 pandemic, online streaming became an essential means of maintaining the connection between sports and their audiences. Broadcasters and digital platforms adapted quickly, providing live coverage of competitions played in empty stadiums (15). Confined to their homes, fans embraced these services as a way to sustain their involvement and continue following teams and athletes (16). Beyond helping the industry endure a period of severe disruption, this transition accelerated a permanent change in viewing practices and highlighted the growing importance of digital media in shaping patterns of fan engagement.

This study investigates the determinants of women's football viewership by analyzing both stadium attendance and online streaming in the Chinese Women's Super League. Drawing on data from the 2023 season, ordinary least squares regression was used to examine attendance demand, while a negative binomial model was applied to streaming viewership. By comparing these two modes of consumption, the study highlights the evolving dynamics of audience engagement in women's football and underscores the importance of digital platforms as complementary to traditional in-person experiences.

Research on the demand for sport has identified a variety of factors that influence stadium attendance across different countries and competitions. Previous studies have proposed multiple ways of classifying these determinants. Borland and Macdonald (17), for instance, grouped them into consumer preferences, economic conditions, quality of viewing, sporting content, and supply capacity. Villar and Guerrero (18) emphasized economic aspects, expected quality, outcome uncertainty, and opportunity costs. Despite these different approaches, most classifications converge on four core categories: competition factors, team-related factors, economic conditions, and match scheduling. These dimensions have been consistently used to explain why spectators attend matches and continue to provide a useful framework for analyzing demand in both traditional stadium contexts and newer forms of digital engagement.

Competition factors are central to understanding sports demand, as the match itself represents the core product. In this sense, sporting contests can be viewed as a form of commodity, with their consumption shaped by both intrinsic characteristics and broader economic conditions (19). The level of excitement generated by a game—through its competitiveness, quality of play, or intensity—has been consistently linked to spectator interest and remains one of the primary drivers of football attendance.

Outcome uncertainty has been a central theme in much of the literature on sports demand (20, 21). The uncertainty of outcome hypothesis suggests that spectators are more attracted to matches where the competitive balance makes the result less predictable (22). However, not all studies support this view, with some reporting little or no association between outcome uncertainty and stadium attendance (21, 23). More recent work has expanded the discussion, questioning whether fans are primarily motivated by the suspense of results or by the broader entertainment value of the contest, such as the number of goals scored (24). Although empirical findings remain mixed, outcome uncertainty continues to be regarded as an important indicator when assessing demand. In addition, goal scoring itself has often been used as a proxy for match quality and has been positively linked to spectator interest (20, 25, 26).

Team-related factors also play a significant role in explaining attendance demand. Studies on Major League Baseball demonstrated that team quality strongly influences spectator numbers (27), while Borland and Macdonald (17) argued that audiences are often drawn to competitions that showcase high levels of skill. More recent research has confirmed that both current performance and historical success continue to affect attendance across different sports. Team loyalty, in particular, exerts a lasting impact on fan behavior, shaped by attributes such as past victories, historical achievements, and seasonal performance indicators like points accumulated (28–31).

Economic factors are another important dimension. Attendance decisions are influenced by both the direct and opportunity costs of attending matches, as well as by broader economic conditions (21, 32). Macroeconomic variables such as market size, population, and income have all been identified as relevant predictors of stadium demand (17, 18). Higher levels of disposable income generally increase the likelihood of attendance, as shown in multiple empirical studies (26, 33, 34), while larger populations expand the potential audience base (35).

Scheduling factors also significantly shape attendance patterns. Games held on weekends consistently attract larger crowds (36–38). Seasonal timing has also been linked to variations in attendance, with some studies noting peaks toward the end of the competitive calendar (30, 37). Prime-time scheduling has similarly been shown to boost viewership by increasing accessibility for spectators (39, 40).

Technological change has further transformed how audiences engage with sport. The expansion of digital technologies and widespread internet access has shifted consumption from traditional stadium attendance to television and, more recently, to online platforms (41, 42). Online viewing via computers, smartphones, and tablets has created new forms of interaction and accessibility (43). Live streaming in particular has grown rapidly, offering personalization, connectivity, and interactivity, which have reshaped the dynamics of the sports industry by enhancing reach and fan engagement.

Much of the existing literature on viewing demand has relied on television ratings as a proxy for audience behavior (44–47). The determinants of TV audiences are largely rooted in the same frameworks used for stadium attendance (25, 32, 48). Building on this foundation, recent studies have begun to analyze demand for online events, which can also be classified into competition factors, team factors, economic conditions, and scheduling.

Pre-game variables, known before matches take place, are particularly important in shaping live streaming demand (17, 18). Among these, outcome uncertainty has received significant attention, though findings are mixed: some studies support the uncertainty of outcome hypothesis (38), while others report inconsistent or no effects (49–54). Goal scoring has also been identified as a strong driver of audience interest, with high-scoring matches associated with greater TV ratings (20).

Beyond competition, team-related variables again play a role in online viewership. Historical records, seasonal performance, and long-term prestige influence digital audiences much as they do stadium spectators (30, 55–57). Scheduling factors are also relevant for streaming audiences, with weekend and prime-time matches attracting higher viewership (36, 37, 56). Finally, the availability of streaming content influences consumption patterns, as videos that remain online longer are more likely to accumulate larger viewing numbers (58).

Together, these strands of research suggest that while traditional attendance and digital viewership share common determinants, the rise of streaming platforms has added new layers of personalization and accessibility that are increasingly important for understanding contemporary sports consumption.

## 2 Materials and methods

### 2.1 Data

Match records from the 2023 season of the Chinese Women's Super League were collected, covering 12 teams and 22 rounds with a total of 132 games. Attendance data were available for 125 matches, obtained from the official websites of the league and individual clubs. These figures served as the dependent variable in the analysis of stadium demand.

Online viewership data were gathered from the league's official Weibo account, which provided real-time live streaming for 126 matches. As no international platforms broadcast these games and unauthorized streams were restricted, the Weibo data offer a reliable measure of digital engagement. All information was collected at the match level, allowing for a direct comparison of determinants across in-person attendance and online consumption. We recorded the cumulative view count displayed on the Weibo live/replay page at the time of data extraction.

### 2.2 Models and analysis

Two regression models were used to analyze the determinants of stadium attendance and online streaming demand. The dependent variables were match attendance and live streaming view counts on the league's official Weibo account, respectively.

#### 2.2.1 Ordinary least squares (OLS) model for attendance

To examine factors influencing stadium demand, pooled cross-sectional data from the 2023 season were analyzed using ordinary least squares (OLS) regression:

$$\mathrm{AT}T_{\mathrm{ij}}=\beta_{0}+\overset{K}{\underset{k=1}{\sum }}\;\beta_{k}X_{k,\mathrm{ij}}+\varepsilon_{\mathrm{ij}}$$

where ATT_ij_ represents the attendance for match *i* between teams *j*, and *X_k_*_, ij_ denotes the set of explanatory variables capturing competition, team, economic, and scheduling factors. Diagnostic tests confirmed the validity of this model. The Breusch–Pagan test indicated no heteroskedasticity (*χ*^2^ (15) = 19.55, *p* = 0.190), and the Breusch–Godfrey test suggested no serial correlation (*p* = 0.815). Variance inflation factor (VIF) values for all predictors were below 10, showing no evidence of severe multicollinearity.

#### 2.2.2 Negative binomial regression for online streaming

Because streaming view counts are count data, several models were considered, including Poisson regression, negative binomial regression, and zero-inflated negative binomial regression (59–61). The negative binomial model was therefore adopted, which accounts for over-dispersed distributions (62, 63).

The dependent variable for the streaming model is WBLIVE (cumulative views); VTIME is included to adjust for differential exposure time of each replay, which mechanically affects cumulative view counts.

The model can be expressed as:

$$\mathrm{In}(E[\mathrm{VIE}W_{i}])=\alpha_{0}+\overset{K}{\underset{k=1}{\sum }}\;\alpha_{k}X_{k,i}$$

Where VIEW*_i_* is is the live streaming view count for match *_i_*, and *X_k, I_* represents the explanatory variables. The dispersion parameter *α* was significantly greater than zero (*α* = 0.407, 95% CI = 0.721–1.058), confirming the suitability of the negative binomial approach. The likelihood-ratio test further indicated model significance [LR *χ*^2^(16) = 86.71, *p* < 0.01].

Although both dependent variables (stadium attendance and streaming view counts) are count data, their distributional characteristics differed considerably. Stadium attendance, after log transformation, approximated a normal distribution with no evidence of heteroskedasticity or serial correlation, justifying the use of OLS regression. In contrast, streaming view counts were highly over-dispersed and strongly right-skewed, with variance far exceeding the mean. Therefore, the negative binomial model was adopted for the streaming data to account for over-dispersion and non-normality.

#### 2.2.3 Model diagnostics

Multicollinearity was assessed for both models. All VIF values were well below the critical threshold of 10, and tolerances exceeded 0.1, confirming stable estimates (64, 65). Based on these results, OLS regression was retained for attendance analysis, while the negative binomial regression model was applied to online streaming demand.

### 2.3 Independent variables

Seventeen independent variables were included in the models, grouped into four categories: competition factors, team factors, economic factors, and match scheduling. The selection of variables followed established frameworks in the literature on stadium attendance, sports broadcasting, and online viewing demand (17, 18, 40, 66, 67). The operational definitions and data sources for all variables are summarized in Table 1.

**Table 1:** List of variables and operational definitions.

Variable	Model		Description
DVs	OLS	NB2	
LATT	O		Natural log of match attendance (log-transformed).
WBLIVE		O	Cumulative online viewing count on the league's official Weibo account at the time of data collection.
IVs			
Competition factors			
SGOAL		O	Sum of both team's goals in the current game
DGOAL		O	Difference between the two teams’ goals in the current game
LTHEIL	O	O	Measure of outcome uncertainty suggested by Theil and Uribe (68) based on the betting odds data times 100
Team factors			
HCHA	O		Home teams’ number of previous CWSL championships won
ACHA	O		Away teams’ number of previous CWSL championships won
CHA		O	Sum of both teams’ number of previous CWSL championships won
SPOINT	O	O	Sum of both teams’ total points until the previous game
DPOINT	O	O	The absolute difference between two teams’ total points until the previous game
Economic factors			
HPOP	O		Home team's local town population
APOP	O		Away team's local town population
POP		O	Sum of both team's local town population
HINCOME	O		Home team's local town per capita disposable income
AINCOME	O		Away team's local town per capita disposable income
INCOME		O	Sum of both team's local town per capita disposable income
Match schedule			
WEEKEND	O	O	Whether the game was held on a weekend (Fri through Sun = 1 vs. Mon through Thurs = 0)
MONTH	O	O	Mar vs. Apr vs. May vs. Aug vs. Sep vs. Nov vs. Dec
VTIME		O	Days elapsed between the replay release and the data-collection date (exposure time).

#### 2.3.1 Competition factors

These variables captured the quality and uncertainty of contests. The sum of goals (SGOAL) and goal difference (DGOAL) measured scoring outcomes. Outcome uncertainty was assessed using Theil's index (68), derived from betting-odds–implied probabilities of home win, draw, and away win. The index was calculated as:

$$\mathrm{LTHEIL}=-100\times \;\underset{k\in \{H,D,\;A\}}{\sum }\;p_{k}\mathrm{lo}g_{10}(\;p_{k})$$

where *p_k_* are the normalized implied probabilities for each outcome. Higher values indicate greater outcome uncertainty (23, 36, 54, 56). This measure is reported as LTHEIL (Theil's index × 100) for readability.

#### 2.3.2 Team factors

Historical and current performance indicators were included to reflect team prestige and competitiveness. The number of championships won by the home (HCHA) and away (ACHA) teams, as well as their combined total (CHA), captured historical success (69). Current season form was measured using the sum of points (SPOINT) and the absolute difference in points (DPOINT) accumulated prior to each match, reflecting both overall quality and competitive balance (22).

#### 2.3.3 Economic factors

Local demographic and financial conditions were measured through population and disposable income. Variables included the home city population (HPOP), away city population (APOP), and their sum (POP), together with per capita disposable income of the home city (HINCOME), away city (AINCOME), and their sum (INCOME). These measures have been widely used to capture the role of market size and economic resources in shaping attendance (17, 18, 26).

#### 2.3.4 Scheduling factors

Match timing was represented by weekend fixtures (WEEKEND), monthly dummies (MONTH), and the number of days a replay video remained available before data collection (VTIME). Prior studies have shown that weekend scheduling, seasonal timing, and replay availability significantly influence both stadium attendance and digital viewership (36–38, 58). In the streaming model, WBLIVE represents the cumulative online viewing count per match on the league's official Weibo account, and VTIME denotes the number of days from the replay's release to the data-collection date, capturing the digital content's exposure window.

## 3 Results

### 3.1 Descriptive statistics

Table 2 presents the descriptive statistics for all variables. Average stadium attendance across the 2023 Chinese Women's Super League was 1,106 spectators per match (SD = 1,535), with a minimum of 60 and a maximum of 11,168. By contrast, the average number of online viewers per game on the league's official Weibo account was substantially higher, at 16,915 (SD = 8,258), ranging from 4,103 to 69,000. These figures illustrate the prominence of digital platforms in extending the reach of women's football beyond the physical limitations of stadiums.

**Table 2:** Descriptive statistics of variables.

Variables	Mean (%)^a^	SD	Min	Max	*N*	Type
ATT^b^	1,106.928	1,535.214	60	11,168	125	Continuous
WBLIVE	16,914.75	8,257.816	4,103	69,000	126	Continuous
SGOAL	2.394	1.755	0	9	132	Continuous
DGOAL	1.788	1.644	0	8	132	Continuous
LTHEIL	42.248	4.935	30.535	47.691	99	Continuous
HCHA	1.5	2.997	0	11	132	Continuous
ACHA	1.583	3.024	0	11	132	Continuous
CHA	3.083	4.060	0	14	132	Continuous
SPOINT	29.283	22.265	0	103	132	Continuous
DPOINT	9.255	9.807	0	39	132	Continuous
HPOP	10.381	6.917	1.147	24.874	132	Continuous
APOP	10.381	6.917	1.147	24.874	132	Continuous
POP	20.762	9.327	3.408	46.732	132	Continuous
HINCOME	7,795.583	2,004.424	5,113	10,970	132	Continuous
AINCOME	7,795.583	2,004.424	5,113	10,970	132	Continuous
INCOME	15,591.17	2,702.765	10,750	21,637	132	Continuous
WEEKEND	0.705	0.458	0	1	132	Dummy
MAR	0.136	0.244	0	1	132	Dummy
APR	0.182	0.387	0	1	132	Dummy
MAY	0.134	0.344	0	1	132	Dummy
AUG	0.182	0.387	0	1	132	Dummy
SEP	0.091	0.289	0	1	132	Dummy
NOV	0.227	0.421	0	1	132	Dummy
DEC	0.045	0.209	0	1	132	Dummy
VTIME	335.833	91.964	202	465	132	Continuous

a Percentage of dummy variables;

b Raw data before log transformation; ATT represents raw attendance before log transformation; LATT (log of attendance) was used in the OLS model.

On-field competition indicators reflected moderate scoring levels, with matches averaging 2.39 goals and a mean goal difference of 1.79. Outcome uncertainty, measured as LTHEIL (Theil's index × 100), averaged 42.25 (SD = 4.94) on a 0–100 scale, with values ranging from 30.54 to 47.69, suggesting notable variability in competitive balance across fixtures. Team performance indicators also showed considerable dispersion, with total points before each match averaging 29.28 (SD = 22.27) and point differences averaging 9.26 (SD = 9.81).

Economic and demographic factors varied widely across host cities. The combined local population of competing teams averaged 20.76 million (SD = 9.33), while the combined per capita disposable income was 15,591 RMB (SD = 2,703). Scheduling variables confirmed that 70.5% of matches were played on weekends. Monthly distributions showed clustering in November (22.7%) and April/August (18.2% each), with relatively few matches in December (4.5%). Finally, replay videos were available online for an average of 336 days, ranging from 202 to 465 days.

Overall, the descriptive statistics highlight clear contrasts between in-person and online consumption. While stadium attendance remained modest, digital platforms attracted significantly larger audiences, underscoring the importance of data-driven approaches to understanding contemporary patterns of engagement with women's football.

### 3.2 Regression results

 Table 3 reports the regression results for stadium attendance (OLS) and online streaming demand (negative binomial model). While both forms of consumption reflect common determinants, their relative importance differs across in-person and digital audiences.

**Table 3:** Results of OLS regression model and negative binomial regression model.

Variables	Dependent variable (OLS): LATT (log of attendance)		Dependent variable (NB2): WBLIVE (cumulative views)		
	Coef.	Robust SE	Coef.	IRR	Robust SE
SGOAL	–	–	0.030	1.030	0.037
DGOAL	–	–	−0.039	0.962	0.038
LTHEIL	−0.774	0.398	−0.860**	0.423	0.464
HCHA	0.090*	0.061	–	–	–
ACHA	0.047	0.062	–	–	–
SPOINT	−0.002	0.010	0.005*	1.006	0.003
DPOINT	−0.019	0.022	−0.014*	0.986	0.008
HPOP	−0.035	0.025	–	–	–
APOP	−0.003	0.027	–	–	–
HINCOME	−0.001**	0.001	–	–	–
AINCOME	−0.001	0.001	–	–	–
WEEKEND	0.759***	0.251	0.119	1.127	0.119
MAR	–	–	−0.341**	0.013	0.849
APR	0.527	0.400	−0.994**	0.018	0.535
MAY	0.477	0.468	−0.786**	0.023	0.469
AUG	0.444	0.519	−0.955**	0.142	0.765
SEP	0.062	0.574	−0.657**	0.191	0.705
NOV	−0.301	0.620	−0.670**	0.497	0.293
DEC	–	–	–	–	–
CHA			0.017	1.017	0.142
POP			0.005	1.006	0.005
INCOME			0.016	1.016	0.247
VTIME			0.017**	1.017	0.007
_cons	11.016***	5.212	9.262***	10,531.66	3.407
*R^2^*	0.396				
Alpha			0.907		
Log pseudolikelihood			−934.660		
LR chi2			86.71***		
*N*	92		94		

Dependent variable in the selection model OLS regression model was *ATT* and the dependent variables of the Negative Binomial regression model were *WBLIVE.*

* *p* < .10,

** *p* < .05,

*** *p* < .01.

#### 3.2.1 Stadium attendance (OLS)

Several variables significantly influenced attendance. Historical success of the home team, measured by the number of past championships (HCHA), was positively associated with attendance (*β* = 0.090, *p* < 0.1), consistent with the role of long-term prestige in sustaining fan loyalty. Scheduling effects were also evident: weekend matches drew significantly larger crowds (*β* = 0.759, *p* < 0.01), reaffirming the importance of convenience and leisure time availability.

Economic context showed an unexpected pattern. Home city income (HINCOME) was negatively related to attendance (*β* = –0.001, *p* < 0.05), suggesting that residents of wealthier areas may have more competing leisure alternatives, reducing the relative appeal of attending women's football matches. In addition, outcome uncertainty (LTHEIL) was negatively associated with attendance (*β* = –0.774, *p* < 0.1), indicating that unpredictable contests did not necessarily encourage fans to attend in person. Other factors—including goal difference (DGOAL), away team championships (ACHA), total points (SPOINT), and population size (HPOP, APOP)—were not statistically significant, suggesting that immediate competitive balance and local demographics played a limited role in determining live attendance.

#### 3.2.2 Online streaming demand (Nb2)

Digital viewership displayed a distinct pattern of determinants. Outcome uncertainty (LTHEIL) was negatively related to streaming demand (IRR = 0.464, *p* < 0.05), while larger point differences reduced viewership (DPOINT, IRR = 0.986, *p* < 0.1), confirming that competitive balance is central to online consumption. Conversely, stronger teams generated greater interest, as the combined points of both teams before the match were positively associated with viewership (SPOINT, IRR = 1.006, *p* < 0.1).

Scheduling effects also extended into the digital domain. Compared with the reference category, matches in March (IRR = 0.849, *p* < 0.05), April (IRR = 0.535, *p* < 0.05), May (IRR = 0.469, *p* < 0.05), August (IRR = 0.765, *p* < 0.05), September (IRR = 0.705, *p* < 0.05), and November (IRR = 0.293, *p* < 0.05) all recorded lower viewership, highlighting the sensitivity of online audiences to seasonal variations. Furthermore, replay availability (VTIME) had a significant positive impact (IRR = 1.017, *p* < 0.05), with each additional day of online availability increasing view counts by approximately 1.7%. Other factors, such as weekend scheduling and local economic conditions, did not show significant effects in the streaming model, suggesting that digital audiences are less constrained by temporal convenience and local demographics.

#### 3.2.3 Comparison

The comparison between models underscores the divergent drivers of in-person and digital engagement. Stadium attendance is influenced primarily by structural and contextual factors—team history, local economic environment, and the convenience of weekend scheduling. By contrast, online streaming demand is shaped more directly by competitive dynamics (outcome uncertainty, point balance, team quality), seasonal timing, and digital accessibility (replay availability). These findings illustrate the complementarity of traditional and digital channels, while also emphasizing the growing importance of digital platforms in broadening the reach of women's football.

## 4 Discussion

### 4.1 Overall findings

This study provides a comparative, data-driven analysis of stadium attendance and online streaming demand in the Chinese Women's Super League (CWSL). By employing ordinary least squares regression for in-person attendance and a negative binomial model for online viewership, the study identified distinct sets of determinants across the two modes of consumption. In-person attendance was shaped by historical success, weekend scheduling, and socio-economic conditions, whereas online streaming was primarily influenced by match competitiveness, team quality, seasonal timing, and replay availability. These contrasting patterns reinforce the argument that digital and traditional channels of engagement are not substitutes but complementary, reflecting the structural transition of sports consumption in the digital era (42, 70, 71).

### 4.2 Stadium attendance

The findings confirm that historical prestige continues to be a powerful determinant of live audiences. The positive impact of home team championships (HCHA) indicates that a legacy of success remains a magnet for fans, echoing previous studies showing that accumulated prestige sustains long-term loyalty (17, 28). This result highlights a challenge for newer or less successful teams, which may need targeted marketing or community-based strategies to compensate for limited historical visibility.

Weekend scheduling was another strong predictor of attendance, with matches held on weekends significantly increasing crowds. This confirms earlier evidence that leisure time availability and convenience strongly influence stadium demand (36). For organizers, this underscores the importance of aligning women's football fixtures with peak leisure periods, ensuring that attendance potential is not undermined by midweek scheduling.

Unexpectedly, per capita income of the home city was negatively associated with attendance. While higher income is generally thought to support greater consumption of sports (26, 72), the substitution effect observed here suggests that residents of wealthier areas may have greater access to alternative leisure options. This finding complicates conventional assumptions and points to the need for pricing and marketing strategies that emphasize the unique live experience of women's football.

Finally, outcome uncertainty exhibited a negative effect on attendance, contradicting the uncertainty-of-outcome hypothesis but consistent with studies that find mixed or weak associations (21, 23). Spectators may be more motivated by the prospect of witnessing dominant performances or high-scoring events rather than uncertain results. This nuance indicates that promotional narratives emphasizing competitive balance may not always align with fan preferences in the context of women's football.

### 4.3 Online streaming demand

Digital audiences demonstrated different consumption logics. Streaming demand increased with matches involving stronger teams (SPOINT), consistent with the appeal of elite-level quality for remote viewers. Conversely, outcome uncertainty (LTHEIL) and greater point differences (DPOINT) reduced streaming demand, suggesting that digital audiences seek both high-quality play and closely contested matches. This finding reinforces earlier evidence that uncertainty and competitive intensity play a stronger role in driving television and digital viewership than live attendance (38, 70).

Seasonal effects were also evident, with matches in March, April, May, August, September, and November showing lower viewership than those in reference months. This seasonal sensitivity may reflect competing sporting events, weather conditions, or academic and work calendars influencing online consumption (44, 45). In addition, replay availability (VTIME) positively affected cumulative views, with each additional day of online access raising viewership by approximately 1.7%. This demonstrates the value of extending the digital lifespan of content, especially in an era where audiences expect flexibility and on-demand access (43).

Unlike stadium attendance, streaming demand was not significantly affected by demographic or economic indicators, nor by weekend scheduling. Digital platforms appear to transcend local market constraints and temporal limitations, offering broader accessibility. This aligns with recent arguments that digital technologies have “deterritorialized” sports consumption, allowing global and asynchronous participation (42).

### 4.4 Implications for women’s football

These findings have several implications for the growth and sustainability of women's football. For stadium attendance, enhancing the profile of historically weaker teams is crucial. Community outreach programs, grassroots integration, and targeted ticket pricing strategies may help mitigate the disadvantage of lacking historical prestige (73). Optimizing scheduling to prioritize weekend fixtures can also increase accessibility and attract more families and young spectators.

For digital engagement, investments in streaming infrastructure and broadcast quality are vital. The sensitivity of digital audiences to competitive balance suggests that production teams should emphasize real-time storytelling around close matches and high-performing teams. Extending replay availability can further capture cumulative audiences, especially among younger viewers accustomed to on-demand consumption (74). These strategies can help women's football harness the expanding digital ecosystem, turning online engagement into a driver of sponsorship and commercial growth.

Importantly, the stark difference between average stadium attendance (∼1,100) and online viewers (∼16,900) underscores the transformative role of digital platforms. Digital consumption now represents the primary avenue for mass engagement with women's football in China, and optimizing this channel is essential for reducing gender disparities in sports media coverage and for broadening the sport's global footprint.

### 4.5 Contribution to sports science and future research

From a sports science perspective, this study advances the literature by demonstrating how digital fan behavior can be integrated into broader models of sports demand. Traditionally, research in biomechanics, physiology, and performance analysis has focused on athlete-centered data, while sports economics has emphasized attendance. By comparing in-person and digital audiences, this study bridges these traditions and highlights the potential of interdisciplinary approaches.

Future research should further integrate audience metrics with athlete performance data collected through wearable technology and biomechanical modeling. For example, combining match-level viewership patterns with GPS tracking, workload monitoring, or injury data could produce a holistic framework linking performance, injury prevention, and fan engagement. Moreover, the application of machine learning to large-scale audience datasets could generate predictive insights into demand fluctuations, supporting real-time decision-making in scheduling, broadcasting, and marketing. Such approaches align with the current trajectory of sports science, which increasingly emphasizes data-driven and technologically integrated methods.

## 5 Conclusion

This study examined the determinants of stadium attendance and online streaming demand in the Chinese Women's Super League (CWSL), providing a comparative perspective on traditional and digital sports consumption. Using ordinary least squares regression for in-person attendance and negative binomial regression for streaming demand, the analysis revealed distinct patterns. Stadium attendance was shaped by structural and contextual factors, including team prestige, local economic conditions, and weekend scheduling, while online streaming demand was driven by competitive balance, team quality, seasonal timing, and replay availability.

The findings contribute to the literature in several ways. First, they expand the study of sports demand to the under-researched domain of women's football, offering empirical evidence from China, a market with growing relevance for women's sports. Second, the results highlight the transformative role of digital media, demonstrating how streaming platforms extend the reach of women's football far beyond stadium capacity and geographic limitations. Third, the comparative approach illustrates how in-person and digital audiences respond differently to sporting events, underscoring the need for integrated strategies that account for both forms of consumption. Finally, the study contributes to broader debates in sports science by emphasizing the potential for integrating fan engagement data with performance metrics, wearable technologies, and artificial intelligence to build holistic, data-driven frameworks for sports development.

In practical terms, the results suggest that boosting stadium attendance requires strengthening the visibility of historically less successful teams and optimizing match scheduling, particularly around weekends. For digital audiences, strategies should focus on enhancing streaming quality, highlighting competitive contests, and extending replay access. Together, these insights underscore the importance of leveraging both traditional and digital channels to promote women's football and to reduce long-standing disparities in exposure and commercial investment.

## 6 Limitations and future research

Several limitations should be acknowledged. First, the dataset is restricted to one season of the CWSL, which limits the ability to identify longer-term patterns or account for inter-season variability. Future research could extend the analysis to multiple seasons or compare across different leagues to provide broader generalizability.

Second, while the models incorporate a wide range of competition, team, economic, and scheduling variables, some potentially important factors were not available. Ticket pricing, stadium accessibility, marketing campaigns, and broadcast production quality could further refine the explanation of both attendance and online demand.

Third, the online viewership data were collected exclusively from the league's official Weibo account. Although reliable, this source does not capture broader digital engagement, such as secondary platforms, international streaming services, or interactive fan behavior through social media. Expanding the scope of digital data could offer a more comprehensive understanding of audience participation.

Finally, the statistical models capture associations but not underlying psychological or motivational mechanisms. Qualitative approaches, survey-based research, or experimental designs could complement quantitative models by providing insights into fan motivations and preferences. Future research may also integrate audience metrics with biomechanical and physiological data obtained from wearable technology or artificial intelligence applications, creating interdisciplinary models that link athlete performance, health, and fan engagement within a single framework.

In addition, future research could integrate both attendance and streaming data within a unified analytical framework. A dummy-coded variable representing the “mode of viewership,” together with its interactions with key predictors, could help assess how the effects of competition, team, and scheduling factors differ between in-person and digital audiences.

## Data Availability

The original contributions presented in the study are included in the article/Supplementary Material, further inquiries can be directed to the corresponding authors.
